# Occupational Health and Safety Issues in Ontario Sawmills and Veneer/Plywood Plants: A Pilot Study

**DOI:** 10.1155/2010/526487

**Published:** 2011-01-03

**Authors:** Dave K. Verma, Cecil Demers, Don Shaw, Paul Verma, Lawrence Kurtz, Murray Finkelstein, Karen des Tombe, Tom Welton

**Affiliations:** ^1^Program in Occupational Health and Environmental Medicine, McMaster University, Hamilton, Ontario, Canada L8N 3Z5; ^2^Ontario Forestry Safe Workplace Association (OFSWA), North Bay, Ontario, Canada P1B 9P1

## Abstract

A pilot study was conducted within the Ontario sawmill and veneer/plywood manufacturing industry. Information was collected by postal questionnaire and observational walk-through surveys. Industrial hygiene walk-through surveys were conducted at 22 work sites, and measurements for wood dust, noise, and bioaerosol were taken. The aim of the study was to obtain data on the current status regarding health and safety characteristics and an estimate of wood dust, noise, and bioaerosol exposures. The occupational exposure to wood dust and noise are similar to what has been reported in this industry in Canada and elsewhere. Airborne wood dust concentration ranged between 0.001 mg/m^3^ and 4.87 mg/m^3^ as total dust and noise exposure ranged between 55 and 117 dB(A). The study indicates the need for a more comprehensive industry-wide study of wood dust, noise, and bioaersols.

## 1. Introduction

Ontario sawmills and veneer/plywood plants are an important part of the Canadian forest industry. The forest industry is an invaluable natural resource and plays an important role in the Canadian economy. The value of forest products exported in 2004 was 44.5 billion dollars with British Columbia, Quebec, and Ontario being the top three contributing provinces. The majority of the forested land in Canada is controlled by the government. They are made up of softwood, hardwood, and mixedwood forests, but the majority is softwood.

**SawMills**

Saw mills process raw logs in a few simple operating steps. Green logs enter the sawmill where they are first debarked and then cut into cants that are further cut into finish pieces of lumber using either circular saws or band saws. Once lumber is cut to size it may be sold as green lumber or may be stacked and dried to specific moisture content through air or kiln drying. Kiln drying involves stacking wood in shed-like structures and ventilating with hot air for 10 to 30 days.

**Veneer/Plywood Plants**

Veneer/plywood plants are more complex than sawmills. Raw logs are debarked, cut to size and heated with steam or hot water. The resulting flitch is rotated on a large lathe and pressed against a long sharp blade to peel off a continuous layer of wood called a veneer. The veneer is cut to size and dried. Sheets of veneer are then sprayed with glue usually phenol-formaldehyde resin or a urea-formaldehyde resin and stacked on top of each other with the grain of wood in an alternating direction and sandwiched in a hot press that forces the pieces together and cures the glue. The ends are then trimmed and the product may be sanded. The standard size for plywood is 4 ft by 8 ft with 3/8 inch thickness being most common.

By-products of wood processing such as wood dust and noise are well known with respect to their occupational health effects. Research on occupational exposure in sawmills and related industries has suggested that workers in sawmills, lumber mills, plywood/particle board factories and veneer plants are at risk of developing allergenic disorders, lung disease and cancer (e.g., asthma, rhinitis, dermatitis, sino nasal cancer, etc.) [1]. 

 Although there is significant information available regarding occupational exposure to wood dust and noise in peer-reviewed published literature from other parts of Canada [2–7] and internationally [8–12], there is almost no peer-reviewed published exposure data available which is from sawmills and veneer/plywood plants of Ontario.

To address this knowledge gap, a pilot study was conducted during 2003–2006 by McMaster University in partnership with Ontario Forestry Safe Workplace Association (OFSWA), an association funded by the Workplace Safety and Insurance Board (WSIB) of Ontario and mandated to provide occupational health and safety services to the Ontario forest industry.

 The purpose of the pilot study was to help in design of a future more comprehensive industry-wide study. The specific objectives were to (i) obtain a snap shot of the current status of sawmills in Ontario regarding industry health and safety characteristics (ii) to have preliminary exposure estimates of prevailing major hazards (i.e., wood dust, noise, and mould) in relation to characteristics of wood (hardwood versus softwood) being processed.

## 2. Materials and Methods

The two research methods utilized were (i) questionnaire survey and (ii) observational industrial hygiene walk-through surveys.

### 2.1. Questionnaire Survey

A questionnaire survey was used to gather information on workplace characteristics, demographics, health and safety practices and opinions/perceptions of employers and employees about occupational health and safety. A listing of sawmill firms was obtained from OFSWA. The names of all sawmill and veneer plants active in 2002 were obtained. Firms were broken down by size (full-time equivalent). Firm sizes in number of workers ranged from 1–5, 6–19, 20–49, 50–59, 110–150, and greater than 150. Four different sets of questionnaires consisting of 26 questions each were prepared—one for (i) health and safety manager, plant manager or owner (ii) joint occupational health and safety management cochair (iii) joint occupational health and safety worker cochair and (iv) health and safety representative. Essentially similar questions were asked in all four sets, with some modest variation. Not all firms received all four sets because only larger firms had joint Health and Safety committees. Larger firms received all four sets while smaller firms employing less than 5 received only one set designed for health and safety manager, plant manager and owner. The questionnaire asked questions regarding (a) workplace characteristics such as type of ownership, how many people work, type of shifts, demography of work force (b) production facilities such as type of mill, what type and % of wood processed, if veneer plant and (c) health and safety at the workplace such as: Is there a hearing conservation program? Is there a respiratory protection program? Is there a medical surveillance program? What type of hazards may be present? The questionnaire for worker cochairs, management cochair and health and safety representative were shorter since it did not ask for workplace characteristics and production facilities. All questionnaires are available to interested readers as a pdf copy from the principal author. 

 According to the OFSWA 2002 year data, there were 363 sawmills and 71 veneer/plywood plants in Ontario employing 15,008 workers. 85% of all firms employed less than 20 workers and 68% employs less than 5 workers. To increase the robustness of data for larger firms (i.e., those with 20 or more workers), we sent questionnaires to all of the large firms; 50% of those employing 6–19 workers and 20% of those employing 1–5 workers. A second mailing was also performed by resending the original questionnaires to all nonrespondent firms in order to increase the response rate.

### 2.2. Observational (Walk-Through) Survey

Onsite visits were made to survey the site conditions, assess various processes for occupational health hazards, consult with site staff (both management and employees) about occupational health and safety issues, validate the responses of the questionnaire surveys and conduct preliminary measurements of airborne dust, noise, mould and take note of potential exposures from other contaminants. Two study investigators conducted the site visits to selected sawmills and plywood/veneer plants in all regions of Ontario except one (north-western region). This exception was made because of the lack of participants (i.e., operating sawmills) in this region due to widespread closure resulting from market downturn. The site visit typically lasted about 6 to 8 hours and comprised of an initial meeting with a company representative, administration of a followup questionnaire and walk-through tour of the facility during which preliminary measurements were made. Generally production process flow was followed from the log yard to the final product staging/packaging.

 Prior to the walk-through survey at each plant, the contact person for the facility who was sent one set of questionnaires was asked to complete the same questionnaire they had previously received in the mail and had completed. Readministration of the questionnaire served to validate the responses given on the original version and helped to identify any changes that may have occurred since the completion of the original questionnaire. Generally re-administration confirmed the earlier responses. This repeat questionnaire was verbally administered by one of the investigators.

 Several parameters were assessed in a preliminary way during the walk-through segment of the visit. This was done in order to characterize the work environment in a preliminary way and it served as a range finding exercise. Twenty-two different site visits from 17 different companies were performed over a three-year period (2003–2006). Due to limited financial resources for this pilot study and the broader mandate of covering a large number of operations from all parts of Ontario, it was decided at the outset to rely on direct reading instrumentation as the method of choice for this range finding exercise. It is hoped that a more comprehensive study will be followed up to this preliminary (pilot) study. Within the resources provided, we would not have been able to cover many sawmills if we were to take a sufficient number of long-term (6–8 hours) personal samples for wood dust, noise and mould at each of the sawmills. 

 Measuring devices used in the walk-through surveys included (i) a real-time direct reading aerosol monitor (DustTrak Mode 8520, Aerosol Monitor, TSI inc. Shoreview, MN, USA fitted with a 10 micrometer (*μ*m) nozzle); (ii) a type 1 sound level meter (Model 2230, Bruel and Kjaer, Denmark); and (iii) a Reuter Centrifugal Air Sampler (RCS) (Biotest AG, Dreieich, Germany). The DustTrak device uses a light-scattering technique to determine mass concentration in real time. When it is equipped with a 10 *μ*m nozzle (impactor), it measures PM_10_ aerosol concentration with an upper particle size limit of 10 *μ*m aerodynamic diameter. Bioaerosol was collected by impaction onto a flexible strip containing 34 agar-filled wells housed in the perimeter of the instrument's impeller head. Bioaerosol laden agar was analyzed for fungal growth and fungal identification in terms of total colony forming unit (CFU/m^3^). The RCS which has an effective air sampling flow rate of 40 liters per minute (lpm) was typically run for one to two minutes at several outdoor and indoor locations.

## 3. Results

The results of four sets of questionnaires and the walk-through surveys are summarized as follows.

### 3.1. Questionnaire Surveys

We received completed questionnaires from 35 firms. Half of the firms completing the survey employed 100 or more full-time and or part-time workers. The numbers of firms by size (i.e., number of employees) returning the questionnaire along with response rates is given in Table 1. The response rates were 33% and 10% for large and small firms, respectively.

According to respondents, both softwood and hardwood species of wood are processed in Ontario. Other salient information from questionnaires are given in Table 2. We asked the participants in the questionnaire survey about the occupational health and safety program. The results show that more than 80% of the employers administer some formal type of hearing conservation program, while about 33% maintain respiratory protection program or a medical surveillance program.

 When asked whether wood dust, moulds, chemicals or noise were workplace hazards, 55 respondents (91%) said yes. Participants were also asked about hygiene monitoring or sampling. 22 said they did monitoring for noise, 18 sampled for wood dust, 10 sampled for chemical exposure and 4 sampled for moulds. In respect to hazard control, the questionnaire results indicate that both natural and mechanical ventilation (general and local exhaust) are used to control hazards of airborne dust and solvent. 

 With respect to hygiene measures related to workplace cleanup, the majority (30 out of 35) use dry sweeping or use compressed air (20 out of 35) to clean floor. In terms of personal protective equipment, the use varied from employer to employer. Some types of respirator was said to be in use by 27 employers (77%). The most common type of respirator used was disposable (*n* = 18) versus half-face cartridge respirator (*n* = 13). Employer and employee health and safety committee members were also questioned about their opinions and the level of risk perception in relation to chemicals, noise, wood dust, mould and other health hazards.

### 3.2. Observational (Walk-Through) Survey

The wood dust measurement by DustTrak with 10 *μ*m nozzle gives results in terms of PM_10_. PM_10_ is mainly used for environmental assessments (outdoor environment). In the occupational setting, size-selective sampling in terms of respirable, thoracic and inhalable samples are the norm [13]. The DustTrak, a direct reading instrument, gives real time measurement and has been used extensively in occupational settings and has proven useful in many workplaces including in an assessment of respirable dust in the construction industry [14]. As stated earlier, the intention was to collect data from a large number of firms and several operations thus we selected to use the DustTrak with 10 *μ*m nozzle as a direct assessment of PM_10_ and an indirect assessment of total dust. Total dust measurement in the occupational setting has been used as the main exposure matrix in the past which is now gradually being replaced in many situations by size-selective sampling of inhalable, thoracic and respirable dust because total dust has no defined acceptance criteria. However, the occupational exposure limits (OEL) in many jurisdictions including Ontario for wood dust is based on measurement by total dust using 37 mm sampler in which airborne dust is collected on a filter contained in a 37 mm diameter closed-face cassette at 2 Lpm. To estimate total dust values from PM_10_ measurement, a conversion between the two is required. Davies et al. [15] conducted a comprehensive study in lumber mills of British Columbia, Canada to determine intersampler ratios for wood dust exposure using inhalable, thoracic (measured by a PM_10_ sampler) and 37 mm total dust sampler. They found intersampler ratio between PM_10_ (using Personal Environmental Monitor (PEM) sampler) used as surrogate of thoracic sample and 37 mm total dust sampler of approximately 1.6. This was based on regression analysis of 24 pairs of samples taken side-by-side where workers wore the two personal samplers (PEM (PM_10_) sampler and total dust sampler). This ratio of 1.6 is somewhat unexpected since total dust normally represents a larger fraction than PM_10_, so the ratio would be expected to be less than 1. It is only the inefficiency of 37 mm total dust sampler versus the PM_10_ sampler used in the sawmill study of Davies et al. [15] that the ratio is greater than 1. We have used this factor of 1.6 as a first degree of approximation to convert DustTrak PM_10_ readings to total dust by dividing the DustTrak readings by 1.6 since this conversion comes from a relevant wood dust industry. We do, however, realize that this ratio of 1.6 may not hold true in other workplaces.

**Figure 1:**
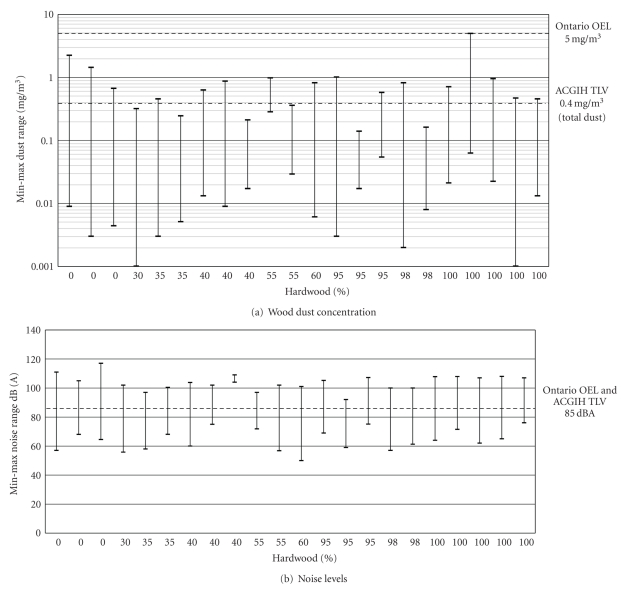
Wood dust concentration and noise levels at 22 sites with respect to % of harwood and softwood use.

**Figure 2:**
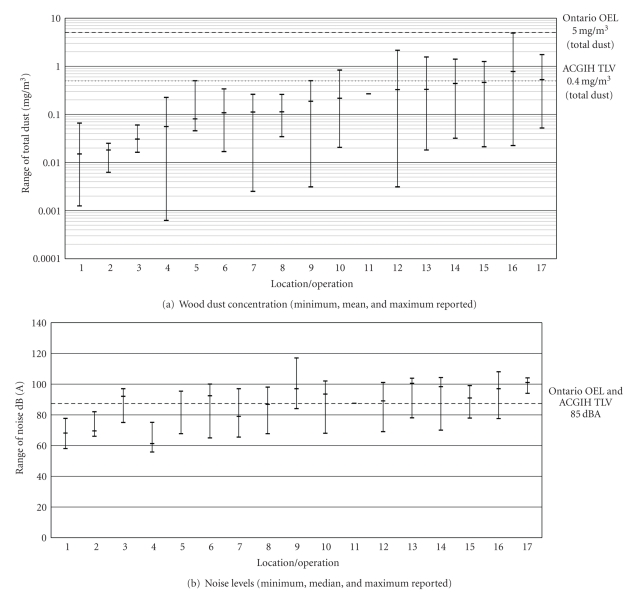
Wood dust concentration and noise levels at 17 location/operations across 22 sites. Location/operation (*n*). 1 = outdoors (13), 2 = log yard (6), 3 = steam room (3), 4 = meeting room (15), 5 = grading (7), 6 = veneer plant (20), 7 = slasher (8), 8 = broadway/greenchain (9), 9 = planer (21), 10 = headrig/sawyer/band saw (24), 11 = bailer (1), 12 = debarker (31), 13 = chipper (14), 14 = trimmer (20), 15 = filing room (7), 16 = edger/stripper (21), 17 = resaw (10), *n* = number of samples.

In Table 3, data from all 22 sites have been summarized in terms of type of mills, type of wood used (hardwood and softwood), range of wood dust exposure as total dust converted from DustTrak PM_10_ reading and range of noise exposure. It is given is ascending order of hardwood use from 0 to 100%. The data is graphically shown in Figure 1(a) in relation to the Ontario Occupational Exposure Limit (OEL) of 5 mg/m^3^ as total dust and also in relation to ACGIH's TLV of 1 mg/m^3^ as inhalable dust converted as an approximate total dust value of 0.4 mg/m^3^ using the conversion ratio of inhalable to total dust of 2.5 to 1 [1]. Data has also been shown for noise (Figure 1(b)) in relation to the Ontario OEL and ACGIH TLV of 85 dB(A) for 8 hrs exposure. The data in Figure 2 is presented by grouping the 17 operations across the 22 worksites where minimum, maximum and mean values of dust concentration (Figure 2(a)) and minimum, maximum and median values of noise exposure (Figure 2(b)) are shown. Results of bioaerosol (fungi) are shown in Tables 4 and 5, respectively.  Table 4 shows the details of eleven fungi identified and quantifies on each of 31 samples. In many samples not all species were present. In Table 5, total colony forming units per cubic meter of air (CFU/m^3^) for all 31 indoor and outdoor samples are listed. More detailed results regarding the questionnaire survey and walk-through surveys at all individual sites as well as copies of the questionnaires can be found in the research report submitted to the funding agency. The report [16] is available from the principal author and can be downloaded from the OFSWA website.

## 4. Discussion

### 4.1. Questionnaire Surveys

35 firms in the Ontario sawmill and veneer/plywood processing industry responded to the questionnaire survey. Although the numbers of questionnaires mailed out were dependent upon the firm size, a large proportion of surveys (45%) were sent to smaller firms (less than 20 workers) which comprise about 85% of the industry. The rate of participation by smaller firms being very low was disappointing. Our response rate for smaller firms of 1–5 and 5–19 workers was around 10% as shown in Table 1. Considering there were over 340 firms in Ontario defined as small business, the low response rate made it difficult to characterize small firms with confidence. In contrast, the response rate for larger firms (20+ workers) was 33% allowing us to draw more accurate conclusions. The industry on average uses an almost even mix of hardwood and softwood species. This contrasts greatly with British Columbia in Canada's forest industry and those in Nordic countries with predominantly softwood use. This has implications regarding wood dust generated from those provinces and countries may not be necessarily reflective for Ontario exposure and thus the need for collection of Ontario-specific exposure data. The Ontario sawmills and veneer/plywood industries employ a large proportion of young workers who by definition are under 25 years of age. Such young workers make up disproportionately large number of workers critically injured according to Ontario Ministry of Labour and WSIB in workplaces (all workplaces, not just sawmills). Although reported widely in the literature as an exposure agent of interest within the sawmills [2, 17, 18] most firms did not report biological agents as being present in the workplace. Only 3 reported using antifungal or biocontrol agent. A greater proportion of firms reported having some form of hearing conservation program (80%) as opposed to respiratory protection/medical surveillance (33%). The reason for this difference could be because hearing protection is regulated under the regulation whereas respiratory protection is only mandatory for certain designated substances.

With respect to occupational health hazard awareness of the industry, 91% recognized at least one exposure factor—wood dust, mould, chemicals or noise as being present in their workplace. Proportionately more workplaces reported to use compressed air or dry sweeping for cleanup which is less desirable compared to vacuum cleaning or wet sweeping. The comparisons of hazard perception between health and safety representatives/worker representatives consistently perceive occupational exposure to be worse than management representatives. This may be due in part to the polar nature of perceptions between labour and management. When asked to rank health and safety compared to other workplace concerns, all of them (both workers and management representatives) rated occupational health and safety as being important to very important.

### 4.2. Observational (Walk-Through) Survey

A wood dust measurement conducted by DustTrak gives only instantaneous readings and not full-shift 8 hours time-weighted average exposure. The result, however, can provide some approximate estimate of likelihood of prevailing full-shift exposure. The measurement of the DustTrak with 10 *μ*m nozzle representing PM_10_ which when converted to total dust taken with 37 mm diameter cassettes with button off also referred to as closed-face cassette system (CFC) provides estimate of total dust. The current wood dust exposure limit in Ontario is in terms of total dust and it is 5 mg/m^3^ for softwood and hardwood but 1 mg/m^3^ for certain hardwood as beech and oak [19]. Generally 5 mg/m^3^ as total dust has been used for wood dust in Ontario. The wood dust exposure as shown in Figure 2(a) ranged from 0.001 mg/m^3^ to 4.87 mg/m^3^ with mean values ranging from 0.06 to 0.78 mg/m^3^. It would thus appear that the total dust level would likely be below 5 mg/m^3^ (the current Ontario occupational exposure limit).

 An exposure study in the Ontario wood-working industries was conducted by a consulting firm for the Government of Ontario in 1986. In this study [20], 23 establishments covering primary, secondary and tertiary wood processing were surveyed including four establishments from primary sector (sawmills and veneer plants). Personal long-term samples were taken for total dust from these four sawmills and veneer plants. They ranged from 0.1 to 6.1 mg/m^3^ (*n* = 37) and two out of 37 samples were greater than 5 mg/m^3^. Unfortunately this study was not published in an open peer-reviewed journal and thus is not readily known or available. Our wood dust data taken by DustTrak and converted to total dust is similar but not directly comparable because our data was not long-term samples.

The trend of relatively lower wood dust exposure in sawmills and primary industries compared to other wood dust exposure sector such as cabinet and furniture making have also been reported recently in a study of inhalable wood dust exposure in the 25 members of the European Union [8] Although the wood dust exposures in Ontario are likely to be lower than the current Ontario occupational exposure limit (OEL) of 5 mg/m^3^ as total dust, but could be in excess of the current adopted threshold limit values (TLVs) of the American Conference of Governmental Industrial Hygienists (ACGIH) of 0.5 and 1.0 mg/m^3^ as inhalable dust [1] and with carcinogenic notation of A1 to A4 which would convert to approximately 0.2 and 0.4 mg/m^3^ total dust using the recommended conversion of 2.5 inhalable/total dust [1] (see Figure 1(a)). There does not appear to be any trend in dust and noise exposure being related to the type of wood used (i.e., higher the % of hardwood usage the higher the exposure) (see Figures 1(a) and 1(b)). Noise levels measured as shown in Figure 2(b) ranged from 55 dB(A) to 117 dB(A). Exposure to noise at many locations are in excess of Ontario noise regulation [21] which is similar to ACGIH-TLV of noise [22] of 85 dB(A) for 8 hours exposure with 3 dB(A) doubling rule. 

 The result of bioaerosols (fungi) listed in Table 5 shows as expected, indoor concentrations to be significantly higher than outdoors. Also there is variations at similar indoor and between different indoor locations. These limited results indicate the need for further study of biological exposures.

## 5. Conclusion

Based on the result of the pilot study, wood dust exposure is expected to be below Ontario's currently regulated exposure limit but it can be in excess of ACGIH current adopted TLV in terms of inhalable dust when converted to approximately total dust concentration. Noise exposure at many operations exceeds the Ontario occupational exposure limit which is the same as ACGIH TLV. Hearing conservation programs need to be instituted especially in the larger firm. 

 The pilot study indicates the need of a comprehensive industry-wide study involving a sufficient number of firms to characterize the wood dust exposure in this industry. A statistically adequate number of personal long-term (full-shift) samples for inhalable and total dusts should be collected. Also some area samples would be useful to obtain. In addition, side-by-side comparison with direct reading devices, such as DustTrak, and long-term samples would be useful for the direct reading devices' use for future exposure assessment in this industry. Biological exposure assessments as part of a comprehensive study would also be valuable. The study shows that larger firms are aware of occupational and safety related issues but need to institute better control programs. On the other hand, we had poor and disappointing responses from smaller firms, so there is a need for focused assessment of smaller firms, since very little is known about the health and safety issues in smaller firms.

## Figures and Tables

**Table 1:** Questionnaire responses by firm size (i.e., number of employees).

Firm grouping	Firm size by # of employees	Number of firms responding
Large*	100+	17
	50–99	4
	20–49	6
Small**	6–9	4
	1–5	4

*82 questionnaires sent out to large firms (response rate = 33%).

*80 questionnaires sent out to small firms (response rate = 10%).

**Table 2:** Salient information from questionnaires.

Question	Response
(i) Type of wood processed	(i) Industry average = 55% softwood and 45% hardwood
(ii) Number of firms using exclusively softwood	(ii) 12
(iii) Number of firms using exclusively hardwood	(iii) 6
(iv) Number of firms reporting as sawmill	(iv) 18
(v) Number of firms reporting as veneer/plywood plant	(v) 17
(vi) Number of firms reporting 25 to 50% workers working unusual work shift (larger than 8 hrs/day)	(vi) 16
(vii) Work force demographics by age	(vii) 25 years or younger = 19% (viii) 35–49 years = 64% (ix) Over 50 years = 17%
(viii) Formal health and safety program	(x) 80% employer administer some form of hearing conservation program (xi) 33% employers have respiratory protection program or a medical surveillance program

**Table 3:** Summary of wood dust concentration as total dust and noise at 22 sites.

Site	Classification	Hardwood %	Softwood %	*N*	Dust max	Dust min	Noise max	Noise min
					(mg/m^3^)	(mg/m^3^)	(dB(A))	(dB(A))
5	Sawmill/planer mill	0	100	25	2.219	0.009	111	57
17	Sawmill/planer mill	0	100	21	1.419	0.003	105	68
20	Sawmill/planer mill	0	100	28	0.669	0.004	117	64.5
3	Veneer plant	30	70	23	0.319	0.001	102	55.8
8	Scrag mill	35	65	11	0.451	0.003	97	58
9	Log mill	35	65	8	0.246	0.005	100.4	68.1
12	Band mill	40	60	14	0.631	0.013	103.8	60
13	Scrag mill	40	60	10	0.856	0.009	102	75
14	Planer mill	40	60	3	0.209	0.017	109	104
6	Scrag mill	55	45	6	0.963	0.278	97	71.8
7	Log mill	55	45	9	0.359	0.029	102	56.8
21	Sawmill	60	40	11	0.824	0.006	101	50
1	Sawmill	95	5	9	1.000	0.003	105.2	69
16	Planer mill	95	5	6	0.139	0.017	92	59
22	Sawmill/planer mill	95	5	18	0.577	0.054	107.2	75.1
2	Plywood/particleboard plant	98	2	32	0.825	0.002	100	57
10	Sawmill/planer mill	98	2	20	0.159	0.008	100	61.3
4	Sawmill	100	0	13	0.700	0.021	107.8	64
11	Sawmill	100	0	8	4.875	0.063	107.9	71.5
15	Plywood plant	100	0	31	0.933	0.022	107	62
18	Sawmill	100	0	8	0.468	0.001	108	65
19	Flooring plant	100	0	9	0.447	0.013	107	76

*N*: number of dust and noise measurements.

**Table 4:** An example of bioaerosols (fungal) concentrations in colony forming units per cubic meter of air (CFU/m^3^) at a location.

Location	Warehouse
Total air volume (Litres)	80
Detection limit (CFU/m^3^)	12.5
Fungal identification:	
* Absidia sp.*	
* Alternaria sp.*	88
* Cladosporium cladosporiodides*	100
* Cladsporium herbarum*	25
* Geotrichum sp.*	—
* Mucor sp.*	13
* Penicillium subgenus Aspergilloides*	86
* Penicillium subgenus Penicillium*	100
* Rhizopus sp.*	—
Sterile mycelium	38
Yeast	88

Total (CFU/m^3^)	540

**Table 5:** Bioaerosol (fungal) concentration in total colony forming units per cubic meter of air (CFU/m^3^) at 15 indoor and 1 outdoor locations.

Locations/operations	*N* (total 31)	Total CFU/m^3^
Indoor		
Chipper room	1	1426
Debarker	6	<LOD; <LOD; <LOD; <LOD; 42; 4625
Dryer	1	<LOD
Edger	1	939
Grading	1	338
Headsaw	1	14175
Lunch room	1	1301
Office	5	<LOD; <LOD; 225; 476; 925
Planer	1	375
Resaw	2	475;1576
Sawyer	1	2700
Stacker	1	400
Storage	1	539
Trimmer	1	1300
Warehouse	1	540
Outdoor		
Outdoors including parking lot	6	<LOD; <LOD; <LOD; 150; 175; 200

*N*: number of samples.

<LOD: less than limit of detection.
